# Trends in the surgical management of proximal humerus fractures in Ireland from 2009 to 2022: An increasing usage of reverse shoulder arthroplasty

**DOI:** 10.1007/s11845-024-03625-5

**Published:** 2024-02-20

**Authors:** Conor S. O’Driscoll, Danilo Vukanic, Tiarnán G. Daly, Diarmuid C. Molony, Petr Jemelik, Eoghan Pomeroy, David E. O’Briain, May S. Cleary

**Affiliations:** 1 Department of Trauma and Orthopaedics, University Hospital Waterford, Waterford, Ireland; 2 Department of Trauma and Orthopaedics, Tallaght University Hospital, Dublin, Ireland; 3 University College Cork, Cork, Ireland; 4 Department of Trauma and Orthopaedics, Royal College of Surgeons, Dublin, Ireland

**Keywords:** Fracture, Open reduction internal fixation, Proximal humerus, Reverse shoulder arthroplasty, Trauma

## Abstract

**Background:**

Proximal humeral fractures are a common injury accounting for a significant workload across orthopaedic departments. Though often managed non-operatively, surgical management is indicated for a proportion of patients.

**Aims:**

The aim of this study is to examine the trends in the management of proximal humeral fractures within Ireland over the past 13 years.

**Methods:**

A retrospective review of Irish Hospital In-Patient Enquiry (HIPE) data was performed between January 2009 and December 2022. Information regarding demographics including age and gender, along with procedure type were collated after patients with proximal humerus fractures, were identified using relevant ICD 10 codes.

**Results:**

Demographic details remained stable with females and those within the 55–69 year age bracket accounting for the highest proportion of patients. The mean annual number of procedures performed across the study period was 365 (273–508), with an increase from 288 cases in 2009 to 441 in 2022. Open reduction and internal fixation were the most common procedures accounting for 76.4% of cases. There has been a rising usage of total shoulder arthroplasty for fixation with an increase from < 5 cases in 2016 to 84 in 2022. A decrease in the usage of hemiarthroplasty and closed reduction internal fixation was also observed.

**Conclusions:**

There has been an increasing volume of operatively managed proximal humeral fractures in Ireland, which sustained despite the 2015 publication of the highly publicised PROPHER trial. The increasing utilisation of total shoulder arthroplasty in acute trauma management is notable and necessitates appropriate training for trauma theatre personnel.

## Introduction

Proximal humerus fractures are a common injury [1] and comprise a large clinical workload across orthopaedic departments nationwide, with an increasing incidence over time [2, 3]. They commonly present as fragility-type fractures following a fall from standing height and have been reported as the third most common fracture type amongst patients over 65 years in the US Medicare population [4]. Though the majority of patients with proximal humerus fractures are managed non-operatively [5], there remains a significant proportion of patients for whom surgical management is indicated. This may be due to a combination of factors relating both to the patient themselves, including their age, activity levels and functional demands, along with the fracture pattern which may have variable displacement, degree of comminution and location which affect the likelihood of successful union [6]. There are a variety of options available to surgeons when considering operative management including open reduction internal fixation with plate and screws or intramedullary nail, closed reduction internal fixation (including percutaneous pinning) and arthroplasty; deciding upon which treatment to offer remains a subject of debate with multiple studies ongoing including the PROPHER 2 trial [7].

The aim of this study is to examine the trends in the surgical management of proximal humeral fractures within Ireland over the period 2009 to 2022. Internationally, there have been significant changes in the management of proximal humerus fractures over this period, with regional variation exhibited [8–11]. This period has also coincided with major external factors which may have affected the delivery of healthcare and surgeon choices including the COVID-19 pandemic from 2020 to 2022. While the flow of traumatic injuries presented to emergency departments remained present despite restrictions [12, 13], there was variation exhibited in the nature of presentations [14] and the number of surgical cases performed in different institutions [15, 16]. Additionally, COVID-related patient anxiety and stress may have been associated with avoidance of in-patient care and operative procedures [17]. The highly publicised PROPHER trial was published during this study period in 2015 which may have impacted upon surgeon decision making [18]. This was highlighted in the UK by Jefferson et al. who performed a survey of 265 surgeons with approximately half reporting a subsequent change in practice [19]. Through information regarding both the volume and breakdown of surgical procedures, this study provides a snapshot of current proximal humerus fracture management in Ireland, which is of interest to both the orthopaedic and broader medical community and essential in healthcare planning.

## Methods

A retrospective review of Irish Hospital In-Patient Enquiry (HIPE) data was performed between the period January 2009 and December 2022. This was facilitated by the Irish National Healthcare Pricing Office who administer HIPE, which is the principal database on inpatient care episodes and discharges from acute public hospitals in Ireland. (HIPE) Patients who underwent surgical management of proximal humerus fractures were identified from within the database using relevant ICD-10 codes for primary diagnosis and Procedure Codes.

This included as follows: primary diagnosis: S4220 (fracture of upper end of humerus, part unspecified), S4221 (fracture of head of humerus), S4222 (fracture of surgical neck of humerus), S4223 (fracture of anatomical neck of humerus), S4224 (fracture of greater tuberosity of humerus) and S4229 (fracture of other and multiple parts of upper end of humerus). Procedure: 4742901 (open reduction of fracture of the proximal humerus with internal fixation), 4742601 (closed reduction of fracture of the proximal humerus with internal fixation), 4891500 (hemiarthroplasty of the shoulder) and 4891800 (total arthroplasty of shoulder—including reverse and anatomical).

Information regarding demographics including age and gender, along with procedure type and volume, were collated in 12-monthly intervals. In line with data protection condition of use, data cells containing five or fewer cases were not specifically reported. Research ethics committee approval was not required for this study, through the usage of an anonymised national database. Descriptive statistical analysis and graphical summaries were used to illustrate our findings. This was performed using Microsoft Excel (Microsoft, Redmond, Washington, USA).

## Results

**Fig. 1:**
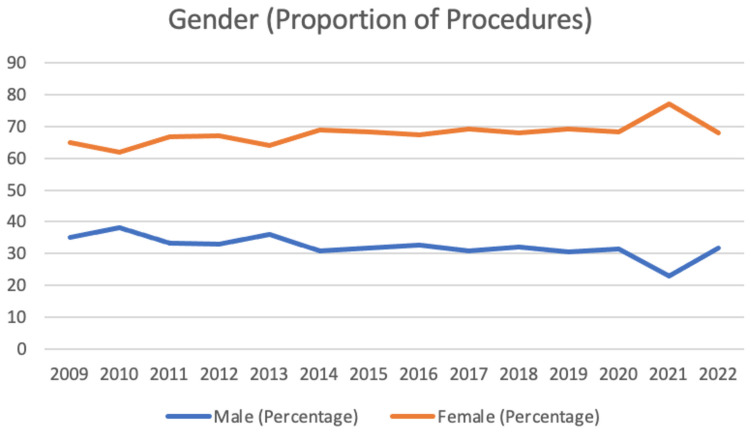
Gender (proportion of procedures)

**Fig. 2:**
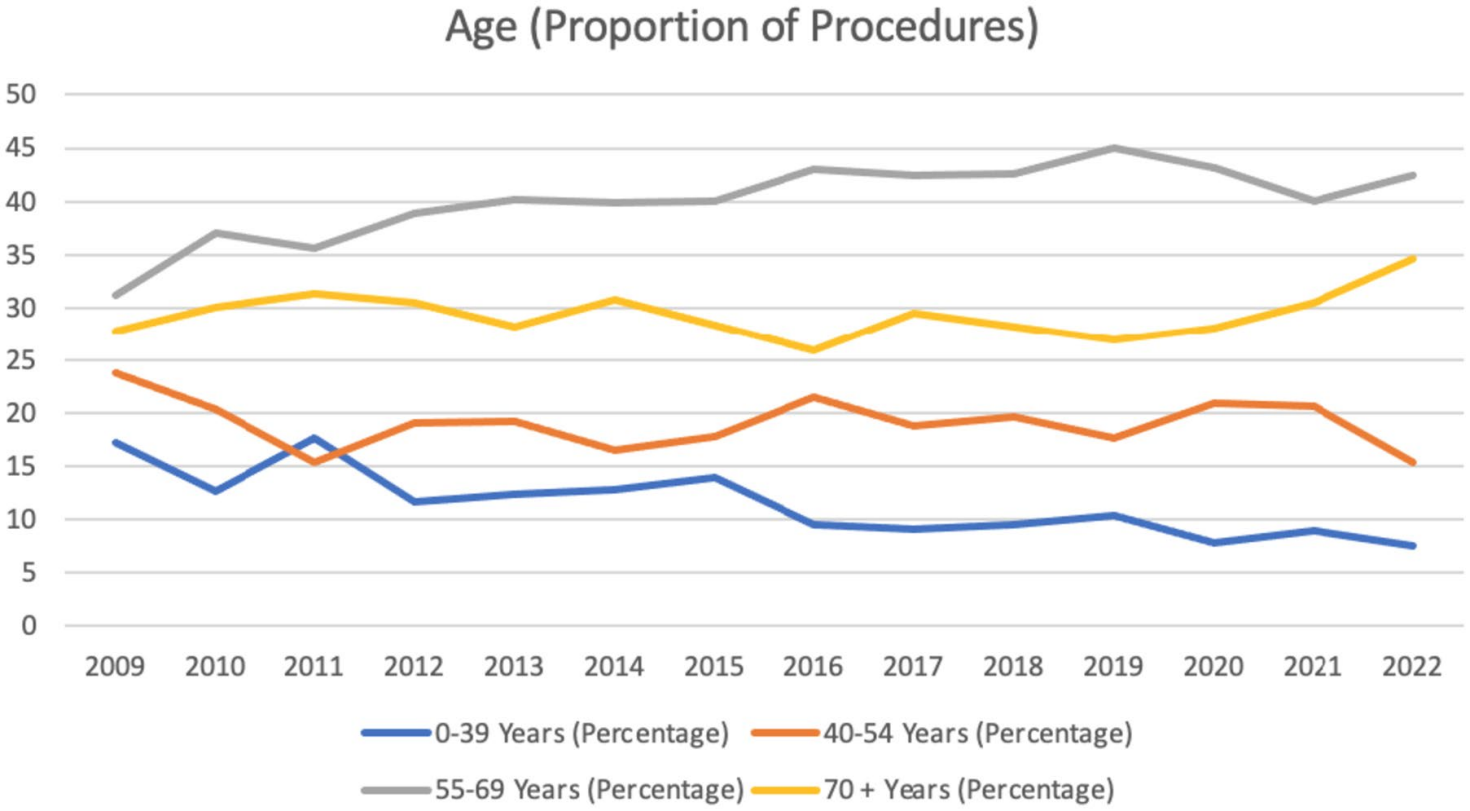
Age (proportion of procedures)

Demographic details remained stable throughout the study period with females, 67.6% (61.89–77.22%) and those within the 55–69 year age bracket 40.45% (31.25–45.01%) accounting for the highest proportion of patients throughout the study period. Further details regarding age groups and gender distribution are provided in Tables 1 and 2 and Figs. 1 and 2.

**Table 1:** Details regarding gender distribution

**Gender (Percentage)**	**2009**	**2010**	**2011**	**2012**	**2013**	**2014**	**2015**	**2016**	**2017**	**2018**	**2019**	**2020**	**2021**	**2022**
Male	35	38.11	33.33	32.88	35.88	30.94	31.65	32.67	30.86	31.99	30.63	31.59	22.78	31.86
Female	65	61.89	66.67	67.12	64.12	69.06	68.35	67.33	69.14	68.01	69.37	68.41	77.22	68.14

**Table 2:** Details regarding age groups

**Age (Percentage)**	**2009**	**2010**	**2011**	**2012**	**2013**	**2014**	**2015**	**2016**	**2017**	**2018**	**2019**	**2020**	**2021**	**2022**
0-39 Years	17.19	12.61	17.65	11.68	12.4	12.87	13.91	9.52	9.14	9.57	10.44	7.83	8.99	7.59
40-54 Years	23.75	20.34	15.36	19.02	19.26	16.58	17.75	21.43	18.86	19.65	17.63	20.87	20.63	15.4
55-69 Years	31.25	36.96	35.62	38.86	40.11	39.85	40.05	43.07	42.48	42.57	45.01	43.19	39.95	42.41
70 + Years	27.81	30.09	31.37	30.43	28.23	30.69	28.29	25.97	29.52	28.21	26.91	28.12	30.42	34.6

**Fig. 3:**
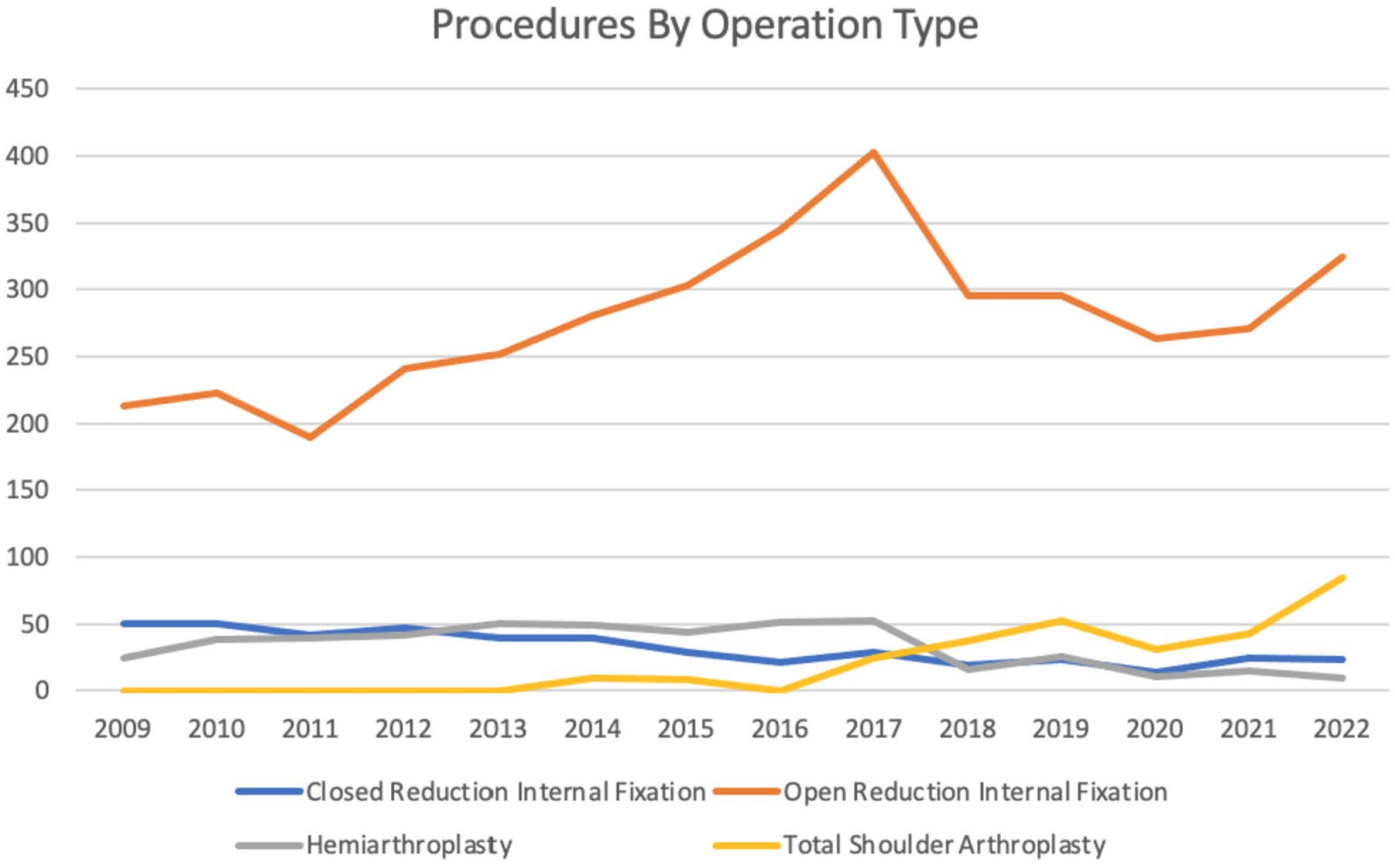
Procedure by operation type

The mean annual number of procedures performed across the study period was 365 (273–508), with an increase from 288 cases in 2009 to 441 in 2022. Open reduction internal fixation was the most common procedure accounting for 76.4% of all cases, with a peak of 82.4% in 2020. The usage of total shoulder arthroplasty increased from < 5 cases in 2016 to 84 in 2022, where it accounted for 19.05% of procedures. A decrease in the usage of hemiarthroplasty, from 25 to 10 (10–50), and closed reduction internal fixation, from 50 to 23 (14–50), was observed between 2009 and 2022. Further details regarding procedure volume are provided in Table 3 and Fig. 3.

**Table 3:** Details regarding procedure volume

**Procedure**	**2009**	**2010**	**2011**	**2012**	**2013**	**2014**	**2015**	**2016**	**2017**	**2018**	**2019**	**2020**	**2021**	**2022**
Closed Reduction Internal Fixation	50	50	42	47	40	39	29	21	29	19	23	14	25	23
Open Reduction Internal Fixation	213	223	190	241	252	281	303	345	403	296	296	263	271	324
Hemiarthroplasty	25	38	39	42	50	49	44	51	52	16	26	11	15	10
Total Shoulder Arthroplasty	0	<5	<5	<5	<5	9	8	<5	24	37	52	31	43	84

## Discussion

The most significant findings from this study were an overall increased volume of operative procedures performed for proximal humerus fractures between 2009 and 2022, with an intermediate decrease between 2017 and 2021. This coincided with an overall 14.35% increase in the Irish population from 4.53 million in 2009 to 5.18 million in 2022. There was a shift in procedure type observed with a trend towards total shoulder arthroplasty in favour of hemiarthroplasty and a decline in closed reduction internal fixation [20].

Similar trends of increasing operative procedures have been observed internationally, though with some regional variation in procedure type. Looking to the USA, Cooke et al. used an aggregated nationwide insurance database between 2010 and 2019, finding an overall increase in the volume of surgically managed proximal humerus fractures [8]. This was attributable primarily to a significant increase in the usage of RTSA which rose 334% between 2010 and 2019, when it was used for 15% of proximal humerus fractures. Compared to our study, there was a greater proportional usage of RTSA compared to ORIF, which was used 60% as much in 2019 as ORIF in Cooke’s US study compared to 25.9% in 2022 in our Irish study. Across other studies, Silva et al. in Portugal from 2000 to 2015 and Jo et al. in South Korea from 2008 to 2016, both reported an increased volume of operative procedures for proximal humerus fractures with early adoption of RTSA [10, 11].

As noted above, there has been a significant increase in total shoulder arthroplasty usage for trauma, which is predominantly reverse total shoulder arthroplasty (RTSA). This trend was first highlighted in the USA, with Alraaba et al. describing an increase year on year from 2010, with a relatively later adoption in Ireland from 2017 onwards [5]. This may also be partly attributable to an increase in the number of fellowship-trained shoulder surgeons experienced in reverse shoulder arthroplasty working in Irish trauma practice during this period, which Hao et al. have shown in a US study to be more likely to perform arthroplasty procedures compared to general trauma surgeons for the similar fracture subtypes [21]. Alraaba et al. described lower reoperation rates compared to ORIF, and other proponents of reverse shoulder arthroplasty in trauma cite higher functional outcome scores and range of motion, particularly in multifragmentary fractures [22–24]. There was a higher readmission rate reported by Alraaba et al.; however, they noted that the RTSA group was older with a greater level of pre-existing medical comorbidities. Though RTSA usage is primarily performed for older age groups, there are increasing studies expanding the usage to younger age groups [25, 26]. Due to relatively low patient volumes, individual procedures could not be reported upon by age group in our study.

The landmark UK PROPHER trial study was published in 2015 [18], during the time period of this study. This was a pragmatic multicentre randomised control trial which compared the surgical and non-surgical treatment of displaced proximal humerus fractures in adults. This study did not demonstrate a significant group difference in Oxford Shoulder Score between pooled surgical and non-surgical treatment. Jefferson et al.’s questionnaire to British Orthopaedic Association and British Shoulder and Elbow Society members returned 265 responses, of whom 137 reported a change in practice to variable extents because of PROPHER, by operating on fewer PROPHER-eligible fractures [19]. However, the response was not uniform [27], with differences in how the study was reported by surgeons [28]. Limitations cited included a failure to differentiate by method of surgical management, and this is a reason for the ongoing PROPHER 2 trial which aims to evaluate reverse shoulder arthroplasty and hemiarthroplasty and non-surgical treatment for older adults with acute 3- or 4-part fractures of the proximal humerus [7]. Within our study, the PROPHER trial did not appear to have an appreciable effect on practice with the number of surgically managed proximal humerus fractures increasing over the years 2015 to 2016 and 2017. A similar high profile UK-based pragmatic randomised control trial was the 2014 DRAFFT study, which compared outcomes of Closed Reduction and Percutaneous Pinning with Open Reduction Internal Fixation for dorsally displaced distal radius fractures [29]. McColgan et al. using HIPE data demonstrated that the DRAFFT study did not appear to influence trends in the management of distal radius fractures in Ireland, with a decreasing usage of percutaneous pinning noted, contrary to the DRAFFT outcomes [30].

The response to the COVID-19 pandemic in Ireland included a number of periods of social restriction and “lockdown”, over 2020 and 2021 [31]. As reported by Hall et al. in an international survey of 185 units, a reorganisation of healthcare services occurred in many hospitals in order to care for patients with COVID-19, and this resulted in reallocation of inpatient areas and reduced operating theatre access for many units along with redeployment of staff including physiotherapists [32]. COVID-related patient anxiety and stress may have been associated with avoidance of in-patient care and operative procedures [17], and together these may explain the reduction in general orthopaedic procedure volume [15, 16] despite continued trauma presentations [12, 13]. Within our study, the volume of operative procedures for proximal humerus fractures was reduced in 2020 and 2021, from previous norms, before rebounding in 2022.

The findings of this study have implications for both training and funding of orthopaedic trauma in Ireland. The increasing usage of newer technologies and procedures such as reverse shoulder arthroplasty which heretofore was predominantly used in elective settings, will require staff training, particularly in hospitals only providing trauma orthopaedic care. This extends not only to operating room personnel but also to surgical trainees [33] and ward staff involved in the patient’s aftercare [34]. There is an increased cost associated with operative management of proximal humerus fractures, which Corbacho et al. estimated to be an additional 1758 Great Britain Pounds in 2016 [35]. This is higher still with reverse shoulder arthroplasty implants which cost significantly more than hemiarthroplasty or plating constructs [35, 36]. This may be offset over the implants’ lifespan; however, through lower reoperation rates and OPD visits, as detailed by Abdel Khalik et al. whom in a Canadian study, concluded that RTSA was a more cost-effective strategy for proximal humerus fractures in older adults than ORIF taking into account additional factors such as readmission, reoperation and Quality Adjusted Life Years [37].

There are a number of limitations associated with this study which are worth acknowledging. As is the case in all studies based on database information, the quality of the information may be subject to human error in coding. The HIPE database collates information based solely on patients admitted to acute hospitals and thus information regarding proximal humerus fractures managed non-operatively is not available as many of these patients would have solely attended outpatient follow up for their injury. It also does not identify patients who may have had deferred procedures such as shoulder arthroplasty performed in elective hospitals following initial conservative treatment or revision procedures. Coding for both Reverse and Anatomic Total Shoulder Arthroplasty is not differentiated; however, one can postulate that the total shoulder arthroplasty procedures performed in the trauma setting are reverse geometry and this assumption was also made by Cooke et al. [8]. There is also no information provided regarding functional outcomes and complications which may affect future care choices.

## Conclusion

There has been an increasing volume of operatively managed proximal humeral fractures in Ireland. Though cases managed non-operatively are not recorded in the HIPE database, it is notable that the number of cases managed operatively continued to rise in the years 2016 and 2017, following the 2015 publication of the widely publicised PROPHER trial. The increasing utilisation of total shoulder arthroplasty in acute trauma management, which heretofore was predominantly used in elective settings, is notable and will necessitate training amongst theatre personnel and equipment procurement, particularly in hospitals only providing trauma orthopaedic care.
